# Analysis of Microbial Diversity Dominating Nitrite Enzymatic Degradation and Acidic Degradation in the Fermentation Broth of Northeast Sauerkraut

**DOI:** 10.3390/foods13244168

**Published:** 2024-12-23

**Authors:** Xiangru Xu, Meng Zhang, Yuefei Tao, Wei Wei

**Affiliations:** School of Agricultural Engineering, Jiangsu University, Zhenjiang 212013, China; xuxiangru@ujs.edu.cn (X.X.); 2212316009@stmail.ujs.edu.cn (M.Z.); 2212416006@stmail.ujs.edu.cn (Y.T.)

**Keywords:** northeast sauerkraut, high-throughput sequencing, prokaryotic microorganisms, nitrite enzymatic degradation, nitrite acidic degradation

## Abstract

Nitrite hazard is an important food safety issue in the production process of Chinese Northeastern sauerkraut, but this nitrite can be eliminated through microbial enzymatic degradation and acidic degradation as fermentation progresses. Therefore, analyzing the microbial diversity that dominates nitrite degradation in Chinese Northeastern sauerkraut can provide a reference for its safe production. In this study, based on the dynamic monitoring of nitrite concentration, pH, and the abundance of nitrite reductase genes (*nirK* and *nirS*) and the application of high-throughput sequencing technology and various statistical analysis methods, the microbial groups associated with nitrite enzymatic degradation and acidic degradation in Northeast sauerkraut fermentation broth were analyzed. During the nitrite peak period of Northeast sauerkraut fermentation broth, the nitrite concentration reached 32.15 mg/kg, the pH was 4.7, and the abundances of the nitrite reductase genes *nirK* and *nirS* were 3.0 × 10^4^ and 4.9 × 10^4^ copies/μL, respectively. At this stage, nitrite degradation was likely dominated by enzymatic activities. Microbial phyla such as *Bacteroidetes* (38.8%), *Proteobacteria* (19.2%), and the archaeal phylum *Euryarchaeota* (1.1%) showed strong correlations with nitrite. Among the genera within these three phyla, *Chryseobacterium*, *Elizabethkingia,* and *Aeromonas* exhibited significant differences in abundance compared to the late fermentation stage and were identified as the primary microbial groups likely driving the enzymatic degradation. During the nitrite degradation period, the nitrite concentration decreased to 0.04 mg/kg, the pH dropped to 3.6, and the abundances of *nirK* and *nirS* genes were reduced to 1.0 × 10^3^ copies/μL. At this stage, the nitrite degradation was primarily driven by acid activity. The bacterial phylum *Firmicutes* (99%) exhibited a strong correlation with pH. Within this phylum, the genus *Lactobacillus*, which showed significant differences in abundance compared to the early fermentation stage, was identified as the primary microbial group indirectly contributing to acidic degradation. This study provides guidance for the isolation of food-grade prokaryotic microbial strains capable of nitrite degradation. Additionally, the findings offer a methodological reference for conducting future research on nitrite-degrading microorganisms in fermented vegetable broths.

## 1. Introduction

Paocai is a traditional Chinese fermented food with strong regional characteristics [1]. However, nitrite hazards often arise during fermentation. Nitrite in paocai is generated through the microbial metabolism of residual nitrogen compounds in the vegetables, and they are subsequently degraded and metabolized by microorganisms as fermentation progresses [2,3,4,5]. The microbial degradation of nitrite in paocai fermentation broth occurs via two mechanisms: direct enzymatic degradation and indirect acidic degradation [6,7]. Enzymatic degradation involves biochemical reactions mediated by nitrite reductase enzymes produced by microorganisms, while acidic degradation is a chemical reaction indirectly driven by the organic acid produced by lactic acid bacteria. The pH of the fermentation broth serves as the boundary indicator distinguishing between these two mechanisms [6]. To date, studies on nitrite degradation in paocai have primarily focused on acidic degradation, and the lactic acid bacteria-based fermentation methods, derived from this theoretical foundation, have already been applied in paocai production [2,8,9,10]. In contrast, research on the enzymatic degradation of nitrites, directly mediated by microorganisms, remains limited. Due to the abundance of lactic acid bacteria and their prominent role in inducing acidic degradation, many researchers have assumed that lactic acid bacteria are also the main microbial group responsible for the enzymatic degradation of nitrites [11]. However, when targeting the genes encoding nitrite reductase for functional gene retrieval in microbial whole-genome databases, such as KEGG, NCBI, and Fungene, we found that classic nitrite reductase genes or homologous genes with more than 30% similarity were absent in the complete genome sequences of common lactic acid bacteria, including *Lactobacillus*, *Weissella*, *Lactococcus*, *Pediococcus*, and *Streptococcus*. These research results suggest that some unidentified non-lactic acid bacteria microorganisms may mediate a self-degradation process of nitrite during the fermentation process of paocai through an enzymatic degradation pathway, together with the nitrite acidic degradation pathway dominated by lactic acid bacteria. Therefore, analyzing the microbial groups involved in nitrite degradation during paocai fermentation—both lactic acid bacteria inducing acidic degradation and non-lactic acid bacteria dominating enzymatic degradation—is crucial for elucidating the microbial mechanisms of nitrite degradation and for controlling the nitrite hazards in paocai production.

However, there are several challenges in studying the nitrite-degrading microorganisms—how to identify and analyze the key microbial communities during the rapid microbial community succession. Low-temperature fermentation can effectively slow down the fermentation of microorganisms, and Chinese Northeast sauerkraut is a suitable model of pickled vegetables that is fermented under low-temperature conditions and has a strong regional characteristic. Northeast sauerkraut, a predominant type of pickled vegetable in Northeast China, is prepared by anaerobically fermenting Chinese cabbage in low-salt brine without any added condiments [12,13]. The process of preparing Northeast sauerkraut involves a gradual decrease in pH and an extended fermentation duration. This unique fermentation profile allows for distinct phases of enzymatic and acidic degradation, making it a suitable model for studying microbial groups involved in both enzymatic degradation and acidic degradation of nitrite in pickled vegetables. However, the absolute dominance of lactic acid bacteria and the limitations in methodologies for studying non-lactic acid bacteria present challenges for detecting the low-abundance non-lactic acid microbial groups. The development of high-throughput sequencing technology offers a feasible solution [14,15,16,17,18]. This culture-independent microbial technique elucidates the environmental microbial diversity, community structure, and functional roles at the DNA level by extracting the environmental microbial genomes [19,20,21]. Compared with the traditional culture-dependent methods, high-throughput sequencing provides a more comprehensive analysis of microbial communities and offers insights into culturable strains. Moreover, high-throughput sequencing technology has been widely applied in the field of food microbiology [22,23,24,25,26]. Therefore, high-throughput sequencing provides robust support for identifying and characterizing non-lactic acid bacterial groups that play critical roles in nitrite enzymatic degradation.

This study integrates the dynamic monitoring of nitrite concentration, pH, and the abundance of nitrite reductase genes (*nirK* and *nirS*) in the fermentation broth of Northeast sauerkraut. High-throughput sequencing technology based on the Illumina platform was employed to analyze the dynamic shifts in microbial community structure associated with changes in nitrite concentration. Diverse statistical analysis methods were utilized to focus on the differential analysis of microbial communities during the nitrite peak and decline phases. By combining the annotation results with microbial whole-genome and functional gene databases, the study identified key microbial groups associated with the enzymatic and acidic degradation of nitrite in the fermentation broth. The findings elucidate the nitrite degradation process during Northeast sauerkraut fermentation and provide guidance for isolating safe, food-grade microbial strains capable of nitrite degradation. Additionally, the results offer a methodological reference for studying the nitrite-degrading microorganisms in the fermentation broths of other types of pickled vegetables.

## 2. Materials and Methods

### 2.1. Preparation of Northeast Sauerkraut

Using a traditional pickling method, Chinese cabbage was blanched with boiling water in an 80 L sterilized paocai jar and then fermented in a low-salt brine (2% NaCl) at 10 °C. The experiment was conducted in triplicate. Each day, a disposable pipette was used to randomly collect 2 mL of fermentation broth from three positions in the middle fermentation layer of each sauerkraut jar. The samples were pooled and centrifuged at 13,780× *g* for 1 min at 4 °C, yielding the supernatant and microbial pellet. The supernatant was immediately used to measure nitrite concentration and pH, while the microbial pellet was reserved for genomic DNA extraction and stored at −80 °C for further use. The nitrite concentration in the supernatant was measured using the N-(1-naphthyl)-ethylenediamine dihydrochloride spectrophotometric method [27]. The pH of the fermentation liquid was measured using a pH meter (pHS-3TC; Shanghai Tianda Instrument Co., Ltd., Shanghai, China).

### 2.2. Extraction of Total DNA, qPCR, and High-Throughput Sequencing

Microbial genomic DNA was extracted on days 1, 3, 7, 9, 11, and 14. TE buffer (100 μL) was added to the extracted DNA, and the mixture was gently shaken to dissolve the pellet. Then, 10 μL of 0.5 M EDTA, 20 μL of 10 mg/mL lysozyme, and 5 μL of 1 mg/mL RNase were added, and the mixture was gently shaken to mix. The sample was incubated in a 40 °C water bath for 30 min as part of the pretreatment process. The microbial genomic DNA from sauerkraut fermentation broth was then extracted according to the instructions provided with the Power Food Microbial DNA Isolation Kit (MoBio Laboratories Inc., Carlsbad, CA, USA).

Samples from the 7th and 14th days of fermentation were processed under the same conditions to extract genomic DNA, following the same procedure. The genomic DNA of *Ochrobactrum anthropi* JCM21032^T^ (which harbors the *nirK* gene) and *Denitratisoma oestradiolicum* JCM12830^T^ (which harbors the *nirS* gene) were extracted. Using these as templates, real-time quantitative PCR assays were established using primers for *nirK* and *nirS*. These primers were used for the quantification of *nirK* and *nirS* genes in the fermentation broth samples.

The extracted DNA from the samples was used as a template for PCR amplification with universal primers for prokaryotes: 515F (5′-GTGYCAGCMGCCGCGGTA-3′) and 909R (5′-CCCCGYCAATTCMTTTRAGT-3′). Takara HS Taq enzyme was used for the PCR amplification, and the reaction system was as follows: 2.5 μL Buffer, 2 μL Mg^2^⁺ (25 mmol/L), 2 μL dNTP, 0.5 μL each of primers F/R, 2.5 μL BSA (5 mg/mL), 0.1 μL Taq enzyme, 1 μL DNA template, and ddH_2_O to a final volume of 25 μL. The reaction conditions were: 95 °C for 5 min (pre-denaturation), followed by 30 cycles of 95 °C for 30 s (denaturation), 55 °C for 30 s (annealing), and 72 °C for 40 s (extension). A final extension step was performed at 72 °C for 10 min. After amplification, PCR products were analyzed by gel electrophoresis on a 1% agarose gel to check the amplification results. The PCR-amplified prokaryotic 16S rDNA from different fermentation periods of sauerkraut fermentation broth was sent to Beijing Novogene Bioinformatics Technology Co., Ltd. (Beijing, China) for high-throughput sequencing. The data were then analyzed to assess the prokaryotic microbial community structure in the sauerkraut fermentation broth.

### 2.3. Bioinformatic Analysis

To obtain more accurate and high-quality DNA sequence information, quality control was performed on the raw high-throughput sequencing data. Chimera sequences were removed using the Mothur software (v1.30.2) [28]. For each obtained OTU (Operational Taxonomic Unit), the species classification information was analyzed using the RDP classifier Bayesian algorithm at a 97% similarity level for the representative OTU sequences. A dilution curve was constructed using Mothur software (v1.30.2). OTU clustering and species classification were based on valid data, and basic analysis results of OTUs and taxonomic profiles for the different DNA samples from sauerkraut fermentation broth at different time points were obtained.

QIIME2 software was used to calculate the Chao1 index, Shannon index, and Simpson index to analyze the abundance and diversity of the obtained OTUs [28,29]. The Chao1 index indicates that higher values reflect greater species richness in the community; the Shannon and Simpson indices reflect the diversity and evenness of the community, respectively. A higher Shannon index indicates greater community diversity, while a higher Simpson index indicates higher evenness.

Species annotation was then performed, and statistical analysis of community structure was conducted at various taxonomic levels. Based on the above data, a series of statistical comparative analyses were performed, including OTU-based clustering analysis, Principal Component Analysis (PCA), and phylogenetic tree analysis, to explore species composition differences and evolutionary relationships between samples.

### 2.4. Statistical Analyses

The statistical analyses included LDA (Linear Discriminant Analysis) to identify species with significant abundance differences between groups, where the length of the bar in the LDA histogram represents the impact of the differential species (i.e., the LDA score). PCA was performed to determine the similarity/differences in the community composition. Canonical Correlation Analysis (CCA) was used to evaluate the correlation between samples and influencing factors, with smaller angles indicating a stronger correlation.

## 3. Results

### 3.1. Dynamic Changes in pH, Nitrite Concentration, Nitrite Reductase Genes, and Prokaryotic Microbial Communities in Northeast Sauerkraut Fermentation Broth

**Figure 1:**
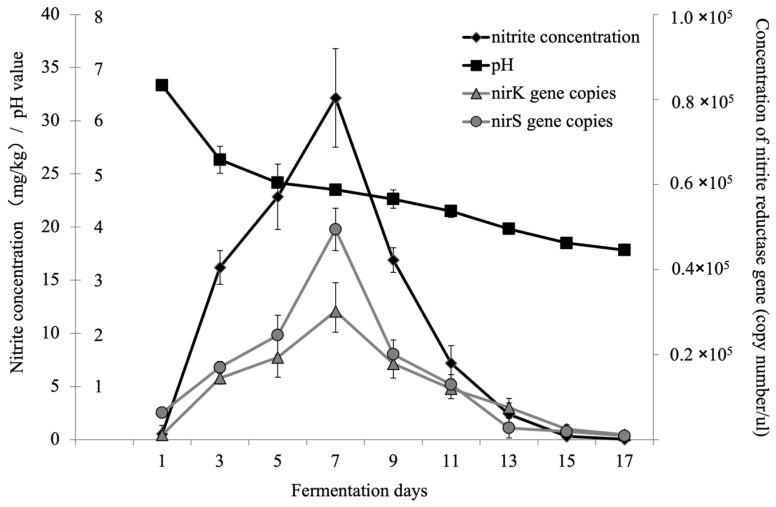
Dynamic changes in pH, nitrite concentration, and nitrite reductase genes (*nirK* and *nirS*) in the fermentation broth.

During the fermentation of Northeast sauerkraut, the nitrite concentration and the abundance of nitrite reductase genes (*nirK* and *nirS*) in the fermentation broth exhibited a similar trend of first increasing and then decreasing, while the pH of the fermentation broth showed a continuous decline (Figure 1). On day 7 of the fermentation, the nitrite concentration in the fermentation broth significantly increased from the initial concentration of 0.55 mg/kg to a peak concentration of 32.15 mg/kg (*p* < 0.05), surpassing the Chinese national standard, which sets the limit at 20 mg/kg. At this point, the abundance of nitrite reductase genes (*nirK* and *nirS*) also reached their peak values, 3.0 × 10^4^ and 4.9 × 10^4^ copies/μL, respectively, while the pH of the fermentation broth significantly decreased from the initial value of 6.8 to 4.7 (*p* < 0.05). As the fermentation continued, the nitrite concentration in the broth gradually decreased, significantly dropping to a level close to the background value (0.06 mg/kg) by day 14 (*p* < 0.05). Meanwhile, the abundance of *nirK* and *nirS* genes gradually decreased to 1.0 × 10^3^ copies/μL, significantly lower than the values during the nitrite peak period and the initial stage (*p* < 0.05). The pH continued to decrease further, reaching approximately 3.6 by day 14.

High-throughput sequencing of the V4 region of the 16S rRNA gene of prokaryotic microorganisms was performed on the Northeast sauerkraut fermentation broth samples collected on days 1, 3, 7, 9, 11, and 14. The quality of the prepared sequencing libraries is shown in Table 1. The number of valid sequences in all samples exceeded 50,000, and the number of OTUs ranged from 66 to 115. Based on these OTU sequences, various indices of the prokaryotic microbial community were calculated, and the results indicated that the microbial communities in the fermentation broth at different stages of fermentation exhibited distinct diversity, evenness, and species richness. Among these stages, the nitrite concentration peak period (day 7) exhibited the highest out number, Shannon index, Chao1 value, and ACE value.

**Figure 2:**
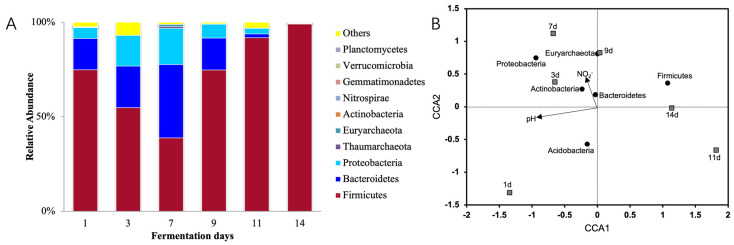
Relative abundance (**A**) and CCA analysis (**B**) of prokaryotic microorganisms in different fermentation stages of sauerkraut.

The OTU sequences of the prokaryotic microbial communities in the constructed libraries were annotated at the species level (Figure 2A). At the phylum level, the results showed that during the initial stage of the fermentation of Northeast sauerkraut, the dominant phylum in the fermentation broth was *Firmicutes*, with a relative abundance of 74.8%, followed by *Bacteroidetes* (16.7%) and *Proteobacteria* (5.7%). As the nitrite concentration increased to its peak (day 7), the relative abundance of *Bacteroidetes* and *Proteobacteria* rose to 38.8% and 19.2%, respectively, while that of *Firmicutes* decreased to 36.8%, which was no longer the dominant phylum. At the same time, the relative abundance of archaea, specifically the *Euryarchaeota* phylum, increased from 0.1% at the initial stage to 1.1%, making it the fourth most abundant prokaryotic phylum. As the fermentation continued, the relative abundance of *Firmicutes* gradually increased. By day 14, when the nitrite concentration decreased to near-background levels, *Firmicutes* became the dominant phylum again, with a relative abundance of 99.0%, while the total relative abundance of *Bacteroidetes*, *Proteobacteria*, and *Euryarchaeota* was less than 1.0%.

A Canonical Correspondence Analysis (CCA) was performed to analyze the prokaryotic microbial community structure at different fermentation stages, the dominant phyla, and factors such as pH and nitrite concentration (Figure 2B). The results indicated that the prokaryotic microbial community structure at the nitrite concentration peak (day 7) was strongly correlated with the nitrite concentration, and the relative abundance of bacterial phyla such as *Bacteroidetes*, *Actinobacteria,* and *Proteobacteria,* as well as the archaeal phylum *Euryarchaeota*, influenced this correlation (Figure 2B). During the nitrite concentration decline period (day 14), the bacterial community structure was more strongly correlated with the fermentation broth pH, and the relative abundance of *Firmicutes* affected this correlation.

Considering the factors such as nitrite concentration, pH, and nitrite reductase gene concentration at the peak (day 7) and decline period (day 14), we hypothesize that during the peak (day 7), nitrite degradation in the fermentation broth was primarily driven by enzymatic degradation, while on day 14, acid degradation became the dominant process. Therefore, based on statistical analysis, a systematic comparison of the prokaryotic microbial communities during the nitrite peak and decline periods will help identify the key microbial groups responsible for nitrite degradation via the enzymatic and acid processes in Northeast sauerkraut fermentation.

### 3.2. Differential Analysis of Prokaryotic Microbial Communities in Northeast Sauerkraut Fermentation Broth During the Nitrite Concentration Peak and Decline Periods

The quality of the high-throughput 16S rRNA V4 region libraries of prokaryotic microbial communities in Northeast sauerkraut fermentation broth on days 7 and 14 is shown in Table 2. Although a higher number of quality sequences were obtained from the day 14 samples, the number of OTUs classified was lower compared to the day 7 samples. The Shannon index, Chao1 value, and ACE value of the day 14 samples were all lower than those of the day 7 samples. These results indicate that during the fermentation period, when the nitrite concentration reaches its peak, the prokaryotic microbial community in the fermentation broth exhibits higher species richness, and the community composition is more diverse and uniform. In contrast, during the later stages of fermentation, when the nitrite concentration returns to baseline levels, the prokaryotic microbial community in the fermentation broth becomes more specialized, with dominant groups exhibiting a more pronounced dominance.

**Figure 3:**
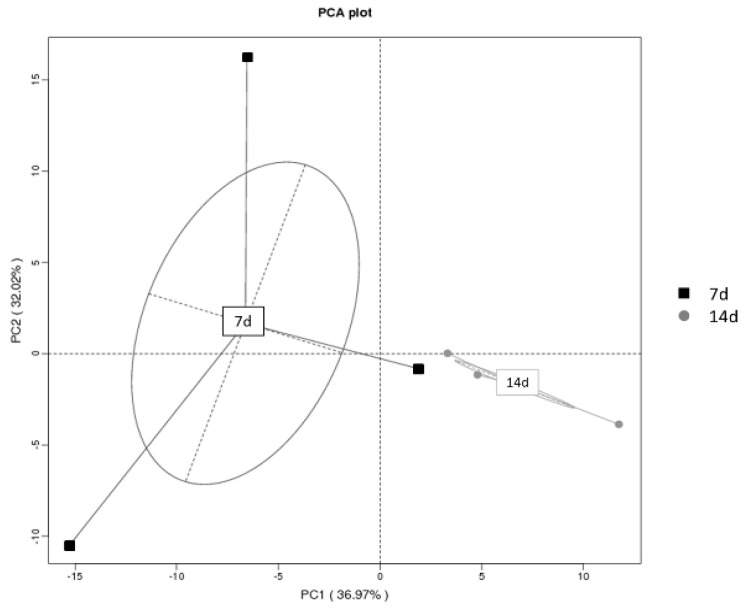
PCA cluster analysis diagram of prokaryotic microorganisms in different stages of sauerkraut fermentation based on OTU analysis.

Principal component analysis (PCA) based on the prokaryotic microbial OTU sequences (Figure 3) showed that during the nitrite concentration peak period (day 7), the PCA distances between different replicates of the sauerkraut fermentation broth samples varied considerably. In contrast, during the nitrite concentration decline period (day 14), the PCA distances between different replicates of the fermentation samples were much smaller. Additionally, after calculating the mean values for the samples from the two periods, the PCA distances were significantly different. This suggests that during the peak nitrite concentration period, the succession of prokaryotic microbial communities in the fermentation broth is highly random, with substantial differences in community composition. However, as the fermentation time increases, the succession of prokaryotic microbial communities tends to become more consistent, with a marked reduction in the differences between replicate samples and a clear contrast from the community composition observed at the nitrite concentration peak period.

**Figure 4:**
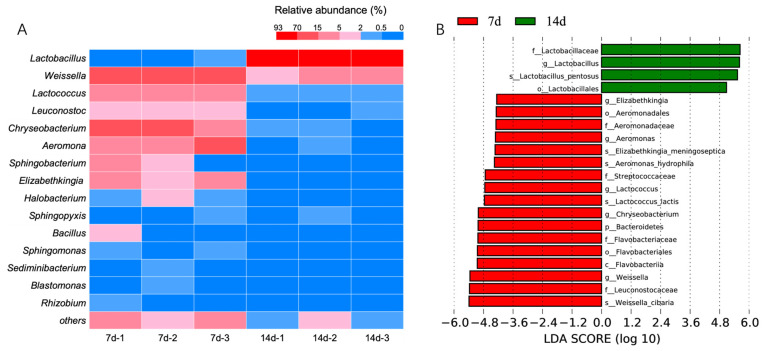
Heatmap (**A**) and the LDA score (**B**) of prokaryotic microorganisms at the TOP15 level from pickles at different fermentation stages.

The species annotation of the prokaryotic microbial OTU sequences from the peak (7d) and decline period (14d) of the nitrite concentration in the Northeast sauerkraut fermentation broth was performed. A clustering heatmap was created based on the relative abundance of the top 15 dominant genera in both periods (Figure 4A), and Linear Discriminant Analysis (LDA) score analysis was conducted to assess the differential impacts on the prokaryotic microbial community structure in the two periods (Figure 4B). The clustering heatmap results showed that during the nitrite concentration peak period, the relative abundance of genera such as *Weissella* (*Firmicutes*), *Leuconostoc* (*Firmicutes*), *Lactococcus* (*Firmicutes*), *Aeromonas* (*Proteobacteria*), *Chryseobacterium* (*Bacteroidetes*), *Elizabethkingia* (*Bacteroidetes*), *Sphingobacterium* (*Bacteroidetes*), and *Halobacterium* (*Euryarchaeota*) was higher compared to the nitrite degradation period. Among them, *Elizabethkingia*, *Aeromonas*, *Chryseobacterium*, *Lactococcus*, and *Weissella* genera showed higher LDA scores, representing prokaryotic microbial groups with significant differences in abundance in the nitrite concentration peak period (7d). In contrast, during the nitrite decline period (14d), *Lactobacillus* in the *Firmicutes* phylum was the only prokaryotic microbial group with higher relative abundance compared to the peak period, and it had a higher LDA score, indicating it was the prokaryotic microbial group with significant differences in abundance in the nitrite decline period (14d). These results suggest that the microorganisms that dominate the microbial community structure during different fermentation periods are significantly different, and as fermentation progresses, the microbial community structure gradually becomes more uniform.

### 3.3. Evolutionary Relationship Analysis of the Prokaryotic Microorganisms in Northeast Sauerkraut Fermentation Broth at Different Periods

**Figure 5:**
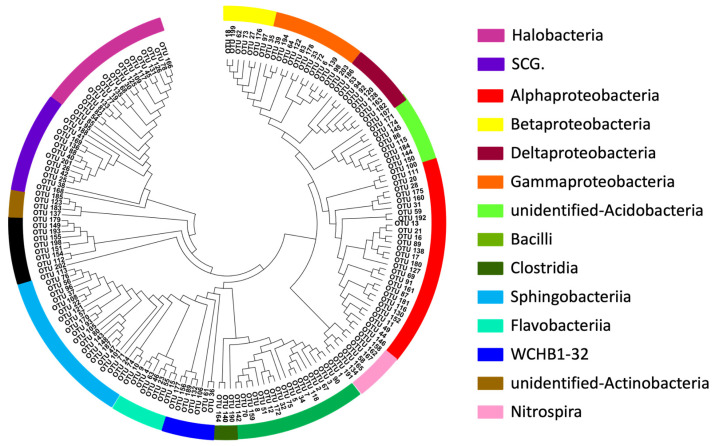
Phylogenetic figure of the OTU sequences from the top 10 prokaryotic microorganism genera in the two stages.

A phylogenetic analysis of the 203 prokaryotic OTUs obtained from sequencing was performed (Figure 5). The three phyla of microorganisms that had the most significant impact on LDA exhibited notable phylogenetic differentiation, with higher OTU diversity. Although the relative abundance and LDA score differences of archaea were relatively low, they also displayed considerable phylogenetic diversity. The OTU sequences from the phyla *Actinobacteria* and *Acidobacteria* could not be annotated at the class level, suggesting that these two bacterial phyla in the fermentation broth contain many microbial groups that have not yet been isolated.

## 4. Discussion

The nitrite degradation process in fermented foods can be divided into two stages: acidic degradation and enzymatic degradation, a concept first proposed by Dodds et al. (1984) in their study of smoked fermented foods [30]. In China, Zhang et al. (2002) simulated the fermentation process using the MARs medium to study the acid and enzymatic degradation of nitrite during vegetable pickling fermentation [31]. They proposed that a pH of 4 serves as the boundary point between the two degradation stages, i.e., when the pH of the fermentation liquid is below 4, nitrite degradation primarily occurs via acidic degradation, and when the pH is above 4, microbial enzymatic degradation becomes the dominant process [32]. The principle of nitrite acidic degradation is that the excess H^+^ produced by lactic acid bacteria in the fermentation liquid reacts with NO_2_^−^, forming unstable HNO_2_, which further decomposes into NO and NO_2_, thus releasing them and thereby degrading the nitrite [6]. The principle of nitrite enzymatic degradation is that microorganisms, under the action of nitrite reductase (Nir) encoded by the *nirK* or *nirS* genes, use NO_2_^−^ as an electron acceptor, forming a respiratory chain to provide energy for themselves, and reduce nitrite to NO, NO_2_, and N_2_, thus degrading nitrite [33]. In this study, the abundance of nitrite reductase genes *nirK* and *nirS* in Northeast sauerkraut fermentation broth reached its peak during the nitrite concentration peak period, indicating the presence of a large number of microbial groups with nitrite reduction potential in the fermentation broth during this period. Considering that the pH of the fermentation broth was 4.8 at this time, we believe that during the nitrite peak period, the degradation of nitrite in the Northeast sauerkraut fermentation broth may primarily occur through microbial enzymatic degradation. However, during the nitrite decline period, when the pH of the fermentation liquid dropped to 3.8, the abundance of *nirK* and *nirS* genes decreased to background levels; these results suggest that the microbial groups with nitrite reduction potential, i.e., the enzymatic degradation microorganisms, gradually disappeared. With the acidic degradation becoming the dominant nitrite control pathway, the nitrite hazard of the Northeast sauerkraut tested was truly controlled. Therefore, the pickled Northeast sauerkraut must be sufficiently pickled for about 15 days; that is, when it is completely in the nitrite acidic degradation stage dominated by lactic acid bacteria, it is the actual safe period for consumption.

To further compare and analyze the microbial communities during the nitrite peak and decline periods, we identified several prokaryotic genera with significant abundance differences in the phyla *Bacteroidetes*, *Proteobacteria*, and *Euryarchaeota* (the latter being Archaea), including *Aeromonas* (*Proteobacteria*); *Chryseobacterium*, *Elizabethkingia*, and *Sphingobacterium* (*Bacteroidetes*); and *Halobacterium* (*Euryarchaeota*). We conducted genome-wide and functional gene (*nirK* and *nirS*) sequence searches for these genera in the NCBI, KEGG, and Fungene databases and found that *Aeromonas*, *Chryseobacterium*, and *Halobacterium* have all been reported to possess the *nirK* gene [34]. Therefore, these three prokaryotic genera may be the primary microbial groups responsible for nitrite enzymatic degradation in the Northeast sauerkraut fermentation broth. Recently, the Chinese paocai industry is promoting the “shallow fermentation” strategy, which means delaying the speed of acidification of the fermentation liquid. This makes the nitrite enzymatic degradation, which has a very short action time, even more important. However, the strains of the key genera in *Aeromonas* and *Chryseobacterium* identified in this study may have conditional pathogenicity characteristics, making it difficult to apply them in practical production. It is worth noting that *Halobacterium*, a haloarchaea, is introduced into the Chinese paocai fermentation system along with salt. Considering the characteristic of haloarchaea that they are extremely prone to absorbing water and swelling to death in low-salt or salt-free liquids, they may be a potentially useful microbial group that can control the nitrite harm through the enzymatic degradation combined with “shallow fermentation” of Chinese paocai. Meanwhile, compared with the haloarchaea dominated by nitrite enzymatic degradation reported previously [33], the haloarchaea detected in this study should be some special groups that are resistant to low salt, and these groups deserve attention in future research.

Detecting the microbial composition at different stages of Northeast sauerkraut fermentation is key to identifying the functional microbes responsible for nitrite degradation. Traditional detection methods are primarily culture-based [35,36,37]; however, they are limited by the number of culturable microorganisms and often underestimate the microbial diversity in the sauerkraut fermentation system due to competition inhibition and the selectivity of culture media [38]. With the continuous development of molecular technologies, early uncultured detection techniques such as clone library methods, denaturing/temperature gradient gel electrophoresis (DGGE/TGGE), terminal restriction fragment length polymorphism (T-RFLP), and real-time quantitative PCR (Q-PCR) have emerged [39,40,41,42,43]. These methods reflect the ecological diversity of microbial communities in sauerkraut fermentation systems at the molecular level. However, the early molecular ecological techniques have limitations, such as an inability to simultaneously perform qualitative and quantitative analysis, low throughput for diversity studies, and limited detection of microbial abundance. The application of these methods in studying the complex microbial groups in sauerkraut fermentation systems is not comprehensive and may overlook certain microbial groups with low abundance that could play important roles. For example, in this study, the archaea *Halobacterium* (*Euryarchaeota*) with nitrite reductase genes has an abundance of <0.1%. The development of next-generation sequencing technologies based on the Illumina platform provides strong technical support for detecting complex microbial systems in sauerkraut [44,45]. This technology enables comprehensive detection of all microbial DNA sequences in the sample’s complex environment, analyzing the diversity and abundance of microbial populations, and more accurately inferring the composition of microbial communities throughout the fermentation process [46,47,48]. Combined with the verification of relevant functional genes and physicochemical properties, this approach can guide the cultivation of potential functional microorganisms for subsequent research [49]. In this study, we utilized Illumina-based high-throughput sequencing technology to sequence sauerkraut fermentation samples at different time points to define the microbial community structure at each stage. By correlating the abundance of *nirK* and *nirS* functional genes with the dynamic changes in nitrite levels, we conduct a detailed analysis of the nitrite degradation mechanisms and related functional microorganisms in Northeast sauerkraut fermentation.

## 5. Conclusions

During the fermentation process of the tested Northeast sauerkraut, the microbial community structure of the fermentation liquid underwent continuous succession, accompanied by the initial increase and subsequent decrease in nitrite content and the abundance of nitrite reductase genes along with the ongoing decline in pH. During the nitrite peak period of the tested Northeast sauerkraut fermentation, the pH of the fermentation liquid was >4.0, and the abundance of the nitrite reductase genes nirK and nirS reached their peak. At this stage, the nitrite degradation in the fermentation broth was likely dominated by microbial enzyme degradation. The bacteria *Aeromonas* (*Proteobacteria*) and *Chryseobacterium* and *Elizabethkingia* (*Bacteroidetes*) exhibited significant differences in abundance compared to the nitrite drop period and likely possessed the nitrite reductase gene *nirK*, making them potential microbial groups responsible for nitrite enzyme degradation during the fermentation process of Northeast sauerkraut. During the nitrite drop period of the tested Northeast sauerkraut fermentation, the pH of the fermentation liquid was <4.0, and the abundance of nitrite reductase genes *nirK* and *nirS* decreased to levels below the initial values. At this stage, nitrite degradation in the fermentation liquid was primarily driven by microbial-mediated acid degradation. The bacterium *Lactobacillus* (*Firmicutes*), which exhibited significant differences in abundance compared to the nitrite peak period, is therefore identified as the primary microbial group responsible for the acid-mediated nitrite degradation during the fermentation process of Northeast sauerkraut.

## Figures and Tables

**Table 1:** Summary of high-throughput DNA sequencing library average values of samples collected from different days.

Sample Name	Original Sequence Number	Effective Sequence Number	OTUs Quantity	Shannon Index	Simpson Index	Chao1	ACEs
1d	79,302	69,927	66	0.82	0.20	69.67	71.84
3d	75,814	57,123	99	2.07	0.68	104.00	103.74
7d	70,227	54,044	115	2.16	0.67	112.27	113.50
9d	89,421	62,491	81	2.04	0.67	79.00	79.36
11d	85,092	68,913	77	1.31	0.44	81.27	82.10
14d	93,839	82,678	80	1.26	0.40	81.56	90.74

**Table 2:** Summary of high-throughput DNA sequencing library average values of samples collected from 7th and 14th day.

Sample Name	Original Sequence Number	Effective Sequence Number	OTUs Quantity	Shannon Index	Simpson Index	Chao1 Index	ACEs
7d	71,361 ± 1342	60,365 ± 864	93 ± 9	1.7 ± 0.2	0.5 ± 0.1	95.3 ± 2.6	96.4 ± 5.3
14d	89,451 ± 3211	71,361 ± 1034	79 ± 13	1.5 ± 0.1	0.5 + 0.1	80.6 ± 4.3	84.1 ± 2.7

## Data Availability

The original contributions presented in the study are included in the article, further inquiries can be directed to the corresponding author.
